# Gangrenous Stomatitis

**Published:** 1870-01

**Authors:** 


### SELECTED ARTICLES.

### ARTICLE VII.

### *Gangrenous Stomatitis.*

HISTORY OF TWO CASES, WITH REMARKS.

**Read before the St Louis Medical Society, by R. S. Anderson, M. D., Demonstrator of Anatomy in the Missouri Medical College.**

During the latter half of the month of May, a general epidemic of measles prevailed at the Protestant Orphan Asylum, in this city. About twenty cases occurred, all of which recovered under the usual expectant treatment now almost universally adopted in this disease. The only sequalæ that followed this epidemic were the two cases of gangrenous stomatitis which I now report.

George Barner, aged 5 years, previously healthy and of seemingly robust constitution, had the measles in an unusually severe form, and did not recover either so completely or so rapidly as the other cases, remaining after the subsidence of the attack in a drowsy, listless condition, with little appetite and severe diarrhœa. The diarrhœa was with some difficulty checked by aperients, followed by astringents, opiates and brandy. Tinc. ferri. chlor, was then freely prescribed, and nutritious food ordered.

About a week after the subsidence of the measles, and after the checking of the diarrhœa, and during the process of general but tardy desquamation of the cuticle, ulcerations were noticed on the mucous membrane of both sides of the mouth, at the point of union of the cheek and gum, opposite the molar teeth of the lower jaw, and partially involving the gum. These ulcerations were at first accompanied by little or no swelling of the tissues of the cheek. There was little or no increase of the salivary secretion. As the ordinary ulcerative stomatitis was then somewhat prevalent in the institution, this was at first supposed to be of that nature, and I think now that the gangrene which followed was grafted upon the ulcerative stomatitis, a fact which, according to Dr. West, seldom occurs. These ulcers were several times thoroughly cauterized with argent. nit., and washes containing chlorate of potash and carbolic acid were alternately and constantly applied locally, and beef-tea, potas. chlor. and iron were ordered freely. Under this treatment the disease rapidly advanced, the cheeks gradually enlarged, became hard and resisting to the touch, and presented externally a diffused red, glistening appearance. The secretion of saliva, which was not at first notably increased, gradually became profuse, the breath fetid, and the molar teeth loose. The ulcers rapidly enlarged at their base, and seemed to be excavating the tissues of the cheek, and were lined with a dark grayish, tenacious, shreddy slough, adhering closely to the subjacent tissue, and difficult of removal by the forceps. The gangrene did not seem to involve the mucous membrane to the same extent as the tissues beneath. During this time the slough was removed, as far as possible, frequently during the day, and sol. carbolic acid freely applied to the surface of the cavity.

On Saturday morning, June 12, I found the left cheek, near the angle of the mouth, perforated by the gangrene, presenting a dark spot in the redness about the size of the end of a lead pencil. At this time the case was seen with me by Drs. Watters and Drake McDowell, and in the afternoon by Dr. Lemoine.

The next day the gangrenous spot had reached the size of a twenty-five cent piece. Assisted by Dr. Quarles, who administered chloroform, I excised the spot, and thoroughly applied nitric acid to its edges and the cavity within, and also to that on the right side, which had not yet perforated.

Up to about this time there had been but little constitutional disturbance observable, but now fever became constant, presenting evening exacerbations. The appetite was increased and even voracious during the whole time, and at no time did the patient complain of any pain. Brandy was administered freely in combination with the other internal remedies. The application of the acid did not seem to affect in the least the spreading of the gangrene. This rapidly progressed, perforated the other cheek, extended above to the lower eyelids, involving both lips and the upper part of the neck, and was finally arrested by the death of the patient on Sunday, June 20. Death seemed to be caused by constitutional contamination, either secondarily to the local disease, or, as I think more probable, primarily causing the gangrene and being itself aggravated and increased by it.

Bertha Lerch, aged four years, German, had measles at the same time, in a milder form, and followed by more rapid recovery, without desquamation. A similar ulceration was observed at the same time as in the former case, (June 1,)

about one week after recovery. It was situated on the posterior portion of the left upper maxillary, on both sides of the last molar tooth. It was treated in a precisely similar manner as the above, without the application of nitric acid, and seemed after a week to be entirely arrested. The cavity in the cheek was small and had ceased to extend; the slough on its surface had disappeared, and the cavity was covered with pus and granulations; the hard firm swelling of the cheek was softening and subsiding. About the 10th of June the last molar tooth, which was loose and caused pain, was removed under my direction by a dentist. On probing the cavity, I discovered necrosis of the alveolar process of the jaw. I flattered myself that the danger in the case had passed. But on Tuesday, June 15, the swelling was observed to be notably increased. The cheek became red, hard and shiny as before, and, on careful examination, I again discovered the dark slough in the cavity.

On Wednesday morning the swelling was still increased, and the disease appeared to have involved the cavity of the antrum Highmorianum. In consultation with Dr. Steele, we applied the pure carbolic acid thoroughly to the cavity in the cheek. I saw this case no more, as it was then removed to the Good Samaritan Hospital, where its mother was a patient. I have since heard, however that the gangrene rapidly extended, perforated the cheek, and ran a more rapid course than the former case, terminating fatally just one week after the perforation of the cheek. Besides the above named gentlemen, I had the benefit of consultation with Drs. Gregory, Bryson, of the City Hospital, and Kealhofer.

I have seen a similar case to these last week at the City Hospital, and have heard of two others occurring in the city at the same time. All of these also proved rapidly fatal.

These cases are reported on account of the variety of the disease, its extreme severity, its rapid progress, and its resistance to all the therapeutic means employed.

The disease is so rare that Dr. West states that he has only had the opportunity of witnessing it in 10 cases, in 8 of which the patients died. Rilliet and Barthez notice 21 cases, of which 20 died. And another French observer, M. Tourdes, has collected from different sources 239 cases which, however, did not all occur in children, of which 176 or 75 per cent., terminated fatally. It is stated seldom to occur in an idiopathic form, but is in almost every instance engrafted upon other affections characterised by grave changes in the circulating fluid. In the large majority of cases, measles is reported as the primary cause, though it has also followed typhoid fever, scarlatina, variola, and intermittent and remittent fever. It has sometimes prevailed endemically in some European countries, among the poorer classes. It seems to be peculiarly a disease of hospitals and institutions in which children are collected together in large numbers, under unfavorable hygienic and dietetic conditions. I think its occurrence in these institutions may be explained by the theory that a large number of cases of contagious disease collected together in a small place may intensify the effect in some of the cases; otherwise I cannot explain the occurrence of cancrum oris in this Asylum, where the conditions for health and recovery are infinitely superior to those existing in the houses from which a majority of the children are brought. It has sometimes, undoubtedly, followed the free administration of mercury. In the two cases reported above, none of this drug had been administered.

There is a difference of opinion in regard to the site of the disease in its incipiency, some authors ascribing it to the mucous membrane, and others principally to the internal substance of the cheek. In the cases above, it undoubtedly originated in the mucous membrane. The majority of cases occur in children between the ages of two and five years.

All authorities agree in the general indications of treatment, and recommend stimulants, nutritious diet, and antiseptics. Much stress is laid upon the early and thorough application of caustic acid to arrest the progress of the slough. This, in the cases reported, did not seem to have the least effect.

The disease, therefore, may be considered as originating in a depravement of the nutritive element of the blood, characterized by the production of a local gangrene, which seems in itself to be possessed of active properties, producing destruction of tissue by mere contact. This process of necrosis is explained by Dr. Bennet as consisting, under these circumstances, in the conversion of the carbon of the tissues into carbonic acid, the hydrogen into water, and the azote into ammonia. The disease called noma, which affects the genitals of young female children, seems to be identical in nature.

The indications deducible from this view are, therefore, two fold: The correction of the systemic contamination, and the improvement of the plastic elements of the blood, and the destruction of the infective properties of the slough. That the treatment directed to the fulfillment of these indications failed in these cases is owing, probably, more to the inadequateness of our therapeutic agents, than to error in their selection.—*Med. Archives.*

---

